# ‘*O*-GlcNAc Code’ Mediated Biological Functions of Downstream Proteins

**DOI:** 10.3390/molecules23081967

**Published:** 2018-08-06

**Authors:** Linhong Zhao, Junaid Ali Shah, Yong Cai, Jingji Jin

**Affiliations:** 1 School of Life Sciences, Jilin University, Changchun 130012, China; yvette0459@126.com (L.Z.); junaid1316@mails.jlu.edu.cn (J.A.S.); caiyong62@jlu.edu.cn (Y.C.); 2 National Engineering Laboratory for AIDS Vaccine, Jilin University, Changchun 130012, China; 3 Key Laboratory for Molecular Enzymology and Engineering, the Ministry of Education, Jilin University, Changchun 130012, China

**Keywords:** post-translational modifications, *O*-GlcNAcylation, OGT, OGA

## Abstract

As one of the post-translational modifications, *O*-linked β-*N*-acetylglucosamine (*O*-GlcNAc) modification (*O*-GlcNAcylation) often occurs on serine (Ser) and threonine (Thr) residues of specific substrate cellular proteins via the addition of *O*-GlcNAc group by *O*-GlcNAc transferase (OGT). Maintenance of normal intracellular levels of *O*-GlcNAcylation is controlled by OGT and glycoside hydrolase *O*-GlcNAcase (OGA). Unbalanced *O*-GlcNAcylation levels have been involved in many diseases, including diabetes, cancer, and neurodegenerative disease. Recent research data reveal that *O*-GlcNAcylation at histones or non-histone proteins may provide recognition platforms for subsequent protein recruitment and further initiate intracellular biological processes. Here, we review the current understanding of the ‘*O*-GlcNAc code’ mediated intracellular biological functions of downstream proteins.

## 1. Introduction

As a ubiquitous post-translational modification, *O*-GlcNAcylation on Ser/Thr residues of proteins in eukaryotic cells is dynamic, inducible, and reversible [1,2,3]. And the dynamic changes of intracellular *O*-GlcNAcylation are controlled by OGT and OGA through adding or removing *O*-GlcNAc group [4,5,6,7]. Numerous studies have demonstrated that *O*-GlcNAcylation is involved in diverse fundamental cellular processes including gene transcription [8,9], cell signaling [10,11], cell cycle progression [12], apoptosis [13], occurrence and development of tumor [14,15]. It is worth noting that *O*-GlcNAcylation occurs exclusively on Ser/Thr hydroxyl groups of nucleocytoplasmic and mitochondrial proteins, leading so to functional responses of target proteins [16,17,18]. Thus, being more and more recognized ‘*O*-GlcNAc code’ may provide recognition platforms or executive instructions for subsequent recruitment of proteins to start the specific biological processes [19]. In this review, we summarize the recent research findings that link crosstalk between *O*-GlcNAcylation and its downstream proteins and speculate on the ‘*O*-GlcNAc code’-mediated intracellular biological functions.

## 2. Add and Remove: Regulation of ‘*O*-GlcNAc Code’ in Cells

So far, only OGT and OGA have been found to be involved in the addition and removal of *O*-GlcNAc groups on Ser/Thr residues of intracellular proteins. As a donor substrate for protein *O*-GlcNAcylation, UDP-GlcNAc is produced by nutrient-dependent hexosamine biosynthetic pathway (HBP) [7,20,21]. In cells, OGT which highly conserved from *Caenorhabditis elegans* to human is responsible for adding *O*-GlcNAc groups to Ser/Thr residues of proteins. Conversely, OGA catalyzes the reverse reaction to remove the *O*-GlcNAc groups from the substrate proteins [6,7]. Three different transcripts of OGT were produced by alternative gene splicing encode respectively, including the nucleocytoplasmic isoform (ncOGT), the mitochondrial isoform (mOGT), and the short isoform (sOGT) [22]. Interestingly, C-terminal of three isoforms is completely the same, and only differs in the number of tetratricopeptide-repeat (TPR) in its N-terminus TPR super-helical structure, resulting in different molecular sizes. The molecular weights of ncOGT, mOGT, and sOGT containing 13.5, 9, and 3 TPRs respectively are 116 kDa, 103 kDa and 75 kDa [22]. In addition to this, a mitochondrial targeting sequence is present in the N-terminal mOGT [22]. The Cat domain contains N-terminal (N-Cat) domains which have residues implicated in catalysis, C-terminal (C-Cat) domains provide binding sites for the sugar donor cosubstrate UDP-GlcNAc and sugar acceptor substrate peptide, and additionaly an intervening Int-d domain [23,24]. Functional domain studies have clarified that each OGT isoform possesses own substrate specificity and functions based on its localizations and the number of TPRs [23,25,26,27]. While they target distinct but overlapping subsets of the proteome [28]. Because of the importance of the TPR domains as scaffold for protein-protein interaction, it would be necessary to precise their role in the modulation of OGT activity [29,30]. In addition, the diversity of OGT function may also be related to its intracellular existence modes. Generally, ubiquitously expressed OGT exists in cells in free forms or assembled in complex to participate in specific intracellular biological processes. For instance, OGT forms complexes with ten-eleven translocationfamily enzymes (TETs) to regulate the enzymatic activity of SET1/COMPASS methyltransferase and gene transcription [31]; forms a heterotrimeric complex with URI (unconventional prefoldin RPB5 interactor) and PP1γ (protein phosphatase 1 catalytic subunit gamma) to play a role in response to metabolic stress [32]; and involves in the composition of NSL (non-specific lethal) complex to regulate histone H4 acetylation [19,33]. On the other hand, OGA is required for cleaving *O*-GlcNAc groups from Ser/Thr residues of substrate proteins. Research reports have revealed that there are two alternative genes splicing OGA isoforms: OGA-L contains 916 amino acids that predominantly localize in the cytoplasm, while OGA-S contains 677 amino acids that localize in nuclear and lipid-droplet [34]. OGA is also composed of N-terminus *N*-acetyl-β-d-glucosaminidase domain and C-terminal pseudo-histone acetyltransferase (HAT) domain [35]. As one of OGT adaptor proteins, OGA can interact OGT and form an “*O*-GlcNAczyme” complex under situation of high glucose [28]. Together, the synergy between OGT and OGA is the key to maintaining intracellular *O*-GlcNAcylation levels. Once this balance is broken, it will lead to abnormal cell function and may even cause cancer.

## 3. ‘*O*-GlcNAc Code’ Provides a Recognition Platform to Initiate Subsequent Functions

Alteration in chromatin structure is tightly associated with gene transcription. Given that DNA and histones are the basic elements that make up chromatin, it is not difficult to speculate that proteins (or protein complexes) that can modify DNA and histones will influence the structure of chromatin and thus regulate gene transcription. Of course, the role of chromatin remodeling enzymes in gene transcriptional regulation by nucleosome sliding and replacement of histone variants cannot be ignored. Recently, accumulating research data reveal that *O*-GlcNAcylation at histones or non-histone proteins can cause changes in nearby histone modifications [36,37,38,39], or lead to the recruitment of specific proteins (or complexes) at local site [31], or increase the accessibility of *O*-GlcNAc-modified proteins to chromatin [31,40], indicating that ‘*O*-GlcNAc code’ may provide recognition platforms for recruitment of subsequent proteins to chromatin, and thereby initiate follow-up biological functions.

### 3.1. ‘O-GlcNAc Code’ on Histones

**Figure 1:**
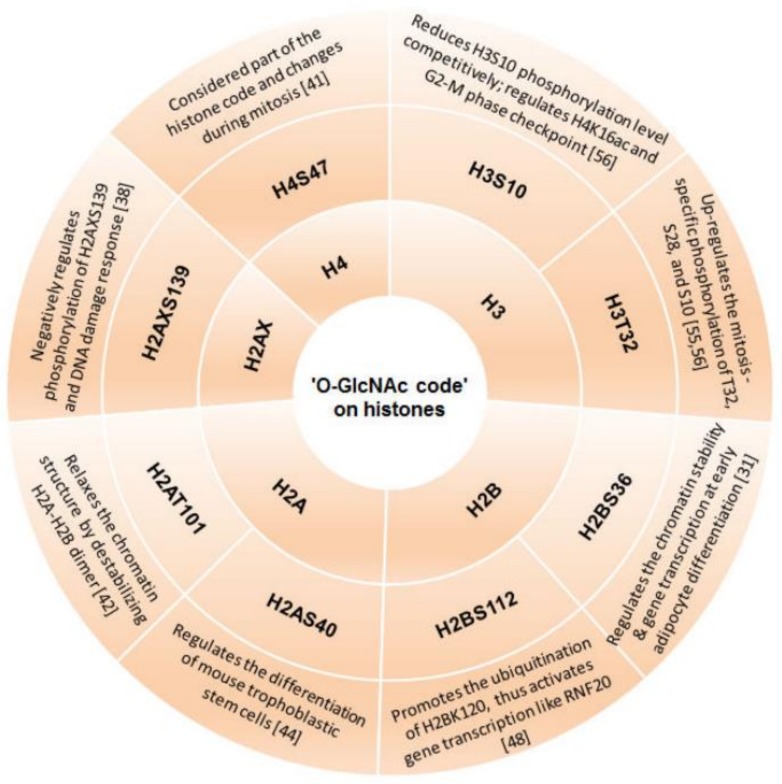
Intracellular biological functions connected to histone *O*-GlcNAcylation. H2AT101, H2A Thr101; H2AS40, H2A Ser40; H2BS36, H2B Ser36; H2BS112, H2B Ser112; H2BK120, H2B Lys120; H3S10, H3 Ser10; H3T32, H3 Thr32; H4S47, H4 Ser47; H2AXS139, H2AX Ser139.

As the basic repeating unit of chromatin, each nucleosome is composed of chromosomal DNA and two copies each of the core histones H2A, H2B, H3 and H4. Each histone N-terminal tail is exposed outside the nucleosomes and can be modified by various enzymes. The modification of histone N-terminal tails such as acetylation, methylation, *O*-GlcNAcylation, etc. directly changes chromatin structure, and thus controlling the activation or inhibition of gene transcription. It is now known that all four histones can be *O*-GlcNAc-modified by OGT in cells [19,36,41]. Interestingly, ‘*O*-GlcNAc code’ on histones can regulate modification of nearby histones. For instance, *O*-GlcNAcylation at Ser112 site of histone H2B (H2BS112) is required for subsequent H2B at lysine 120 (H2BK120) mono-ubiquitination [37]. This ubiquitination may provide a recognition platform for recruiting histone H3K4 methyltransferase SET1/COMPASS, and further activates gene transcription [31]. In adipocytes, ‘*O*-GlcNAc code’ on H2BS112 maintains chromatin stability at the early stage of cell differentiation, consequently results in restraining gene transcription in cell fate [42]. Moreover, ubiquitination of H2BK120 is involved in ring finger protein 20 (RNF20)-mediated DNA double strand breaks (DSBs) repair [43]. However, the accurate interplay between *O*-GlcNAcylation and mono-ubiquitination needs further explored. Subsequent analysis of mass spectrometry identified several *O*-GlcNAcylation sites on histones including H2A Thr101 (H2AT101) [42], H2B Ser36 (H2BS36) [31], and H4 Ser47 (H4S47) [41]. ‘*O*-GlcNAc code’ on those sites changes during mitosis, regulates histone tail dynamics, and further alters chromatin structure [41,42], demonstrating the involvement of ‘*O*-GlcNAc-code’ in cell cycle regulation and gene transcription. Interestingly, genome-wide distribution of *O*-GlcNAc code at Ser40 of histone H2A during the differentiation in mouse trophoblast stem cells is changed dramatically, implicating the important role of *O*-GlcNAcylation in the differentiation of stem cells [44]. Histone *O*-GlcNAcylation-mediated intracellular biological functions are as shown in Figure 1.

### 3.2. OGT Assembled in Different Protein Complexes to Coordinate Specific Functions

Alterations of chromatin structure in cells are often accomplished by multiprotein complexes including chromatin-modifying or chromatin remodeling enzymes. As mentioned before, intracellular OGT is frequently assembled as a component into protein complexes, and coordinate with other subunits to perform the corresponding biological functions. The TETs are good examples. TET proteins including TET1, TET2, TET3 are mainly responsible for catalyzing the conversion of 5-methylcytosine (mC) to 5-hydroxymethylation (hmC) [45]. Recent data have verified that each enzyme of the TETs can be complexed with OGT to initiate their subsequent functions. First, the binding of OGT to TET1 proteins increases its chromatin accessibility by stabilizing its own protein [31,45]; Second, the complex of OGT and TET2/3 recruit protein complexes to chromatin to regulate subsequent gene transcription. For example, OGT-TET2/3 complex can recruit SET1/COMPASS complex by *O*-GlcNAcylation of the host cell factor 1 (HCF1), a subunit of the SET1/COMPASS complex, therefore promoting catalytic activity of H3K4 tri-methylation (H3K4me3) by SET1/COMPASS [31]. In contrast, recruiting PCR2/Ezh2 (enhancer of Zeste 2 polycomb repressive complex 2 subunit, a subunit of PRC2 complex) and Sin3A (SIN3 transcription regulator family member A)/HDAC1 (histone deacetylase 1)/SIRT1 (sirtuin1) complexes to chromatin by OGT-TET1 complex repress gene transcription [10,46,47]; Third, OGT together with TET2 regulate histone H2BS112 *O*-GlcNAcylation in embryonic stem cells [48]. Overall, OGT proteins in collaboration with TETs play a critical role in regulating chromatin structure and gene transcription.

OGT can also form complexes with proteins other than TETs in cells. We previously identified MOF (males absent on the first)-containing histone acetyltransferases NSL (non-specific lethal) complex, which is responsible for acetylating histone H4 at lysine 16 (K16), 5 (K5), and 8 (K8) [33]. In our experimental conditions, OGT directly binds to and stabilizes NSL3 (a subunit of the NSL complex) through *O*-GlcNAcylating NSL3, in turn affecting the activity of H4K16ac, H4K5ac, and H4K8ac by NSL complex [19]. In fact, HCF1 is also one of the subunits of the NSL complex [33]. It has been reported that *O*-GlcNAcylation by OGT to HCF-1 at the Thr11 site on proteolysis repeat domain may provide instructions for the HCF-1 proteolysis [49]. Furthermore, OGT/HCF1 subcomplex stabilizes PGC-1α, a master regulator of gluconeogenesis, suggesting the role of OGT/HCF1 in gluconeogenesis [50]. According to Burén et al., a complex formed by OGT/URI/PP1γ regulates *O*-GlcNAcylation mediated metabolic stress [32]. Although the precise function is unclear, it is a fact that OGT, HCF1 and THAP1 can form a stable complex [51]. Considering that THAP1 is a sequence-specific DNA binding factor, we speculate that this complex may play a role in the regulation of gene transcription. There is still a similar case that OGT cooperates with deubiquitinase USP7 to regulate MLL5 protein stability by *O*-GlcNAcylating it, thus prevents MLL5 from being degraded [52].

### 3.3. Function Switching between O-GlcNAcylation and Phosphorylation

On account of the fact both *O*-GlcNAcylation and phosphorylation occur on Ser/Thr residues of substrate proteins, extensive crosstalk between *O*-GlcNAcylation and phosphorylation through mutual inhibition of the same or nearby residues are studied [53]. For example, nuclear proteins are highly *O*-GlcNAcylated during interphase, but become phosphorylated during mitosis [54]. More specifically, higher levels of *O*-GlcNAcylation on histone H3 reduce mitosis-specific phosphorylation at Ser10, Ser28, and Thr32, suggesting the mechanistic switch during the cell cycle. [55]. It is worth noting that histone H3S10 has been considered as a molecular checkpoint for efficient entering mitosis, on the other hand, Aurora kinase B (an essential regulator of mitotic progression) is responsible for phosphorylating H3S10 [56], indicating the involvement of Aurora kinase B in the functional switching between *O*-GlcNAcylation and phosphorylation. In another case, OGT-dependent *O*-GlcNAcylation is often enriched at DNA damage foci, and negatively regulates DNA damage response. Especially, *O*-GlcNAc-modified H2AX at S139 and MDC1 (DNA damage check point 1) are detected at DNA damage foci, and negatively regulates DSB-induced phosphorylation at Ser139 of H2AX [38]. Histone *O*-GlcNAcylation-mediated intracellular biological functions are as shown in Figure 1.

The crosstalk between *O*-GlcNAcylation and phosphorylation is also reflected in histone deacetylase HDAC1. In hepatocellular carcinoma (HCC) cells, *O*-GlcNAcylation of HDAC1 at Thr114 and Ser263 in turn promotes its phosphorylation, thereby further impacts its deacetyltransferase activity [57]. Occasionally, OGT may temporarily build complexes in respond to certain reactions. For example, OGT and OGA form a transient complex at M phase with PP1γ and Aurora B together to regulate the phosphorylation status on vimentin [16]. Furthermore, ‘*O*-GlcNAc code’ on numerous kinases drastically regulates the downstream signaling pathways through functional switching altering modification status between *O*-GlcNAcylation and phosphorylation. For instance, *O*-GlcNAcylation at Ser189 of CamKIV (Calcium/calmodulin-dependent protein kinase IV) inhibits its phosphorylation at Thr200, thereby blocks its ATP binding ability and phosphorylation catalytic activity [10]. As an important signaling mediator, Glycogen Synthase Kinase 3β (GSK3β) is involved in circadian clock regulation [58]. It has been reported that the activity of OGT can be regulated by GSK3β through phosphorylating OGT at Ser3 or Ser4. Interestingly, OGT itself can also be *O*-GlcNAcylated on the same or neighboring serine residues, suggesting the coordinative functions between phosphorylation and *O*-GlcNAcylation events on OGT itself [59]. Similar restrictions between *O*-GlcNAcylation and phosphorylation are also reflected on a microtubule-associated Tau protein. *O*-GlcNAcylation of TauS400 specifically inhibits phosphorylation at S404 of Tau by CDK2/cyclinA3 kinase. In contrast, phosphorylation of neighboring S396 and S404 can decrease TauS400 *O*-GlcNAcylation [60]. In summary, *O*-GlcNAcylation and phosphorylation may be indicative of functions in rapid switching between different functions.

### 3.4. ‘O-GlcNAc Code’ on Non-Histone Proteins Targets Specific Biological Functions

In cells, OGT mediated *O*-GlcNAcylation frequently targets transcriptional regulators including transcription factors/co-factors, chromatin structure remodelers to regulate certain gene transcription [61,62]. As reported by Howerton et al. [63], OGT as a promising placental biomarker is implicated in maternal stress and reprogramming of CNS gene transcription in development. In breast cancer MCF-7 cells, ‘*O*-GlcNAc code’ on Ezh2 at Ser75 stabilizes the PCR2 complex, which further targeting histone H3 to catalyze the tri-methylation of lysine 27 (H3K27Me3) [64]. Deserve to be mentioned, *O*-GlcNAcylation on CARM1 (co-activator-associated argine methyltrnsferse 1) at Ser595, Ser598, Thr601 and Thr603 is essential for its substrate specificity, and *O*-GlcNAcylation of CARM1 regulates the methylation of histone H3 at arginine 17 (H3R17) [65,66]. *O*-GlcNAcylation of MLL5, a specific histone H3K4 trimethylase, restrains the expression of histone variant H3.3, thus promotes self-renewal progression adult glioblastoma cells [52,67,68]. In another case, *O*-GlcNAcylation of histone chaperone HIRA at S231, S425, S593 and S878 by OGT incorporates histone variant H3.3 into genetic regions, then further controls H3.3 nucleosome assembly and cell senescence [69,70,71]. Likewise, ‘*O*-GlcNAc code’ on T457 of DNA polymeraseη (Polη) enhances genome stability [72,73].

‘*O*-GlcNAc code’ mediated regulation of intracellular biological functions is also involved in the signaling pathway. As we all know, signal transducer and activator of transcription 3 (STAT3) is a key mediator of intestinal inflammation. ‘*O*-GlcNAc code’ on Thr717 of STAT3 negatively regulates its phosphorylation and targets gene expression in macrophages, suggesting the importance of *O*-GlcNAcylation on colonic inflammation and inflammation-associated diseases [39]. The effect of ‘GlcNAc code’ on the AKT (protein kinase B) signaling pathway is also extremely important. It is clear that AKT signal pathway promotes survival and growth in response to extracellular signals [74]. For example, a rapid increase of *O*-GlcNAcylation at both Thr308 and Ser473 of AKT resulted by cerebral ischemia promotes neuronal apoptosis through down-regulating AKT activity, suggesting a negative correlation between AKT phosphorylation and *O*-GlcNAcylation in ischemic stress response [74]. β-catenin, a subunit of the cadherin protein complex, acts as an intracellular signal transducer in the Wnt signaling pathway [75], demonstrating the importance of protein stability of β-catenin in maintaining this Wnt signaling pathway. Fortunately, *O*-GlcNA modified sites at the N terminus of β-catenin (S23/T40/T41/T112) has been identified by ETD-MS/MS. Theses modification stabilizes β-catenin through direct competition with phosphorylation at T41 [76], although the precise mechanism needs further exploration. In addition, SIRT1 as a vital stress sensor contributes in but not limited to stress responses, metabolism, genome stability maintenance and ageing [77,78]. Under genotoxic, oxidative, and metabolic stress stimuli, OGT directly binds to and *O*-GlcNAc-modifies C-terminus of SIRT1 at Ser549, so that enhances SIRT1 deacetylase activity and promotes cell survival, strongly suggesting the role of OGT/*O*-GlcNAcylation-mediated cytoprotection of SIRT1 [79].

## 4. ‘*O*-GlcNAc Code’ Mediated Diseases

Based on OGT is an important and indispensable enzyme in cell metabolic pathway, it is not difficult to understand that the imbalanced *O*-GlcNAc level leads to the occurrence of many diseases including diabetes, neurologic disorders, cardiovascular disease, and cancer [80].

### 4.1. ‘O-GlcNAc Code’ in Cancer

Tumor suppressor p53 (also known as p53) has been described as “the guardian of the genome“ because of its role in conserving stability by preventing genome mutation [81]. Under normal conditions, p53 is ubiquitously degraded through ubiquitin-proteasome-mediated degradation pathway [82]. However, when the cells encounter various stimuli, p53 is stabilized and activated by *O*-GlcNAcylation at Ser149 residue in response to stress [11]. Similarly, in cancer cells, alteration in *O*-GlcNAc homeostasis regulates p53 stabilization and translocation to the nucleus, and transcriptional activity of p53 is increased by acetylated p53 at Lys382, therefore, leads to gene expression of p53 target genes such as p21. Interestingly, stabilization of p53 by *O*-GlcNAcylation is not seen in p53 hypermutated ovarian cancer cells [83]. Nevertheless, evidence reveals that the depletion of mutant p53 attenuates malignant degree of cancer cells, suggesting that mutant p53 stability may promote the acquisition of new pro-oncogenic activities including cell proliferation and metabolic changes. Thus, mutant p53 will provide an attractive druggable target and novel approaches for cancer therapy [84], providing the evidence that the expression of p53 wild type is a potential cancer therapy approach. In some cases, although specific sites for *O*-GlcNAcylation on certain proteins have not yet been discovered, the overall *O*-GlcNAcylation indeed alter its downstream biological function. For example, *O*-GlcNAcylation-mediated SIRT1 regulates the stability of oncogenic transcription factor FOXM1 in a MEK/ERK-dependent manner in breast cancer cells [85]. While in prostate cancer and breast cancer cells, the OGT/*O*-GlcNAcylation-mediated stabilization of c-MYC is tightly associated with the cell growth, suggesting the possibility of c-MYC as a potential upstream regulator of OGT target genes [86,87]. However, reducing global OGT/*O*-GlcNAcylation in gastric cancer cells increases PUMA and caspase-3, and further promotes cell apoptosis, suggesting that OGT is required for cell growth and survival in gastric cancer [88]. In addition, *O*-GlcNAcylation of NF-κB is involved in nuclear translocation of NF-κB which subsequently activates the matrix-metalloproteinases (MMPs) transcription that the key protease enzymes facilitating metastasis of cholangiocarcinoma (CCA) [89].

### 4.2. ‘O-GlcNAc Code’ in Diabetes

Diabetes mellitus (DM), commonly referred to as diabetes, is a complex metabolic disorder with high blood sugar levels (hyperglycemia) over a prolonged period. Two major types of diabetes including Type 1 (T1DM) and Type 2 (T2DM) are classified according to the absolute or relative lack of insulin signaling [90]. Numerous actors of insulin signaling including the intracellular subunit of the insulin receptor, IRS1 and IRS2, PDK1, the protein kinase Akt/PKB, and the transcription factor FoxO1 can be modified by *O*-GlcNAcylation [91,92,93,94,95]. And in most cases, ‘*O*-GlcNAc code’ on those proteins has effects opposite to those induced by insulin [91]. For example, elevated *O*-GlcNAcylation act as an intermediate in the insulin signal pathway, reducing the insulin-stimulated phosphorylation of Akt2 and IRS-1, thereby subsequently leading to insulin resistance in rat primary adipocytes [92]. On the other hand, ‘*O*-GlcNAc code’ on Akt at Thr305 and Thr312 disrupt the interaction between Akt and PDK1, in turn inhibit the phosphorylation of Akt at Thr308 [93]. In addition, insulin secretion by the β-cell is regulated by a transcription factor NeuroD1 through controlling the binding activity with OGT. Under high glucose conditions, NeuroD1 interacts with OGT, on the contrary, combines with OGA. *O*-GlcNAc modified NeuroD1 easier to localize in the nucleus to increase binding to DNA and glucose-dependent insulin synthesis [94]. Similarly, hyper-*O*-GlcNAcylation of pancreatic/duodenal homeobox-1 (PDX-1) also enhances its DNA binding to the A-box in the HR2 region of FFA1 promoter (free fatty acid receptor-1), thus stimulating insulin secretion [95]. What’s more, the significant increase of *O*-GlcNAc modification in erythrocytes of diabetic and pre-diabetic patients are observed, revealing that *O*-GlcNAc levels may be a biomarker for estimating the efficiency of treatment and early detection of diabetes, thus providing valuable information for clinical diagnosis and treatment [96,97]. Despite this, roles of *O*-GlcNAc modified proteins in the progression of pancreatic dysfunction still need further exploration.

### 4.3. ‘O-GlcNAc Code’ in Alzheimer’s Disease

Alzheimer’s Disease (AD) is a chronic neurodegenerative disease with a decrease in regional glucose metabolism [98]. Declined glucose metabolism in the brain often induced by amyloid-β peptide (Aβ) toxicity, and T2DM leads to low levels of brain *O*-GlcNAc [99]. Forebrain-specific loss of *O*-GlcNAcylation results in progressive neurodegeneration, including widespread neuronal cell death, neuro inflammation, increased production of hyper phosphorylated tau and amyloid genic Aβ-peptides, and memory deficits in adult mice [100], indicating the involvement of *O*-GlcNAcylation in the progression of AD. *O*-GlcNAc modification on a microtubule-associated protein tau is another important factor that implicated in the progression of AD [101,102]. Currently, four *O*-GlcNAc sites including Thr-123, Ser-208, Ser-400, and Ser-409/Ser-412/Ser-413 on human tau have been mapped [103,104]. Even so, the detailed mechanism of *O*-GlcNAc modified tau needs to be further explored. Proteomic analysis of mouse synaptosomes show about 20% of synaptosome proteins to be *O*-GlcNAcylated, and ‘*O*-GlcNAc code’ on those proteins are implicated in processes like neurite growth, axonal branching, synaptic plasticity, and mitochondrial trafficking [105,106,107,108,109]. For instance, hyposensitivity to thermal stimuli, lose epidermal innervation, and axonal outgrowth deficits in culture are observed in OGT-knock-out mice [110], suggesting that OGT/*O*-GlcNAcylation is essential for survival of sensory neuron and target innervation.

### 4.4. ‘O-GlcNAc Code’ in Cardiovascular Diseases 

Sometimes OGT/*O*-GlcNAcylation acts as an alarm or stress signal. In response to various forms of stress such as ischemia, hypoxia, oxidative, osmotic, ultraviolet light, and others, a transient increase in *O*-GlcNAc level is frequently occurred [111]. For example, when myocardial ischemia-reperfusion injury occurs, global levels of *O*-GlcNAcylation are augmented in response to stress stimuli [112,113]. Owing to this, increased *O*-GlcNAcylation and OGT expression during heart failure in mice are observed [114], suggesting that protein *O*-GlcNAcylation as vital compensatory mechanism may be required for pressure overload hypertrophy and infarct-induced heart failure [115]. Based on these data, *O*-GlcNAcylation could be effectively considered as mediator of cardioprotection. However, in diabetes, the chronically elevated *O*-GlcNAcylation level is closely associated with dysfunction, leading at terms to diabetic cardiomyopathy. Thus, *O*-GlcNAcylation could also have detrimental effects for heart function and cardiomyocyte OGT is necessary for maturation of the mammalian heart [116,117].

According to the OGT and OGA are the only enzymes that involved in *O*-GlcNAc post-translational modification, it can be speculated that the two enzymes should play a critical role in the basic biological processes in cells. Because of this, the imbalanced *O*-GlcNAcylation is widely involved in many diseases.

## 5. Conclusions and Perspectives

**Figure 2:**
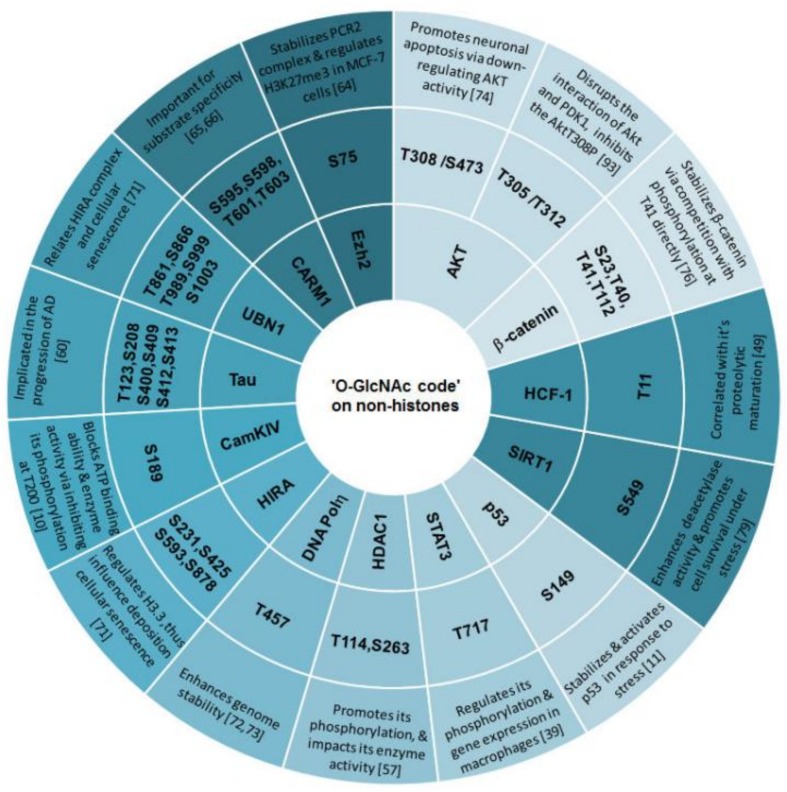
‘*O*-GlcNAc code’ mediated biological functions of downstream proteins. T and S in the figure represent Threonine and Serine. For example, S75 represents Serine 75.

*O*-GlcNAcylation on histones or non-histone proteins is one of the most common protein post-translational modifications (PTMs). Regardless of whether the ‘*O*-GlcNAc code’ on histones or non-histones, *O*-GlcNAc-modification is widely involved in varies cellular biological processes through regulating cellular metabolism, chromatin stability, and gene transcription. Importantly, ‘*O*-GlcNAc code’ on substrate proteins often significantly influences nearby histone or non-histone modifications, protein biological functions, protein stability, intracellular localization, or provides a recognition platform for subsequent protein recruitment and further initiates specific functions of downstream proteins (Figure 2). Therefore, finding the *O*-GlcNAc site on the substrate proteins seems to be extremely important for exploring the specific functions of downstream proteins. With the development of high-throughput protein profiling technology, the *O*-GlcNAcylation sites on substrate proteins are continuously discovered. In contrast, the exact function of its downstream proteins and its network with other proteins or pathways in cells appear to be lagging behind. Especially, OGT/*O*-GlcNAcylation integrates *HBP* metabolic pathway and thereby widely modulates basic biological functions. Thus, clarifying the functional coordination between ‘*O*-GlcNAc code’ and its downstream proteins will provide a theoretical basis for follow-up research.

## Abbreviations

*O*-GlcNAc, *O*-linked β-*N*-acetylglucosamine; OGT, *O*-GlcNAc transferase; OGA, glycoside hydrolase *O*-GlcNAcase; HBP, hexosamine biosynthetic pathway; TET, ten-eleven translocation; PP1γ, protein phosphatase 1 catalytic subunit gamma; Ser/Thr, Serine/Threonine; SIRT1, sirtuin1; HDAC1, histone deacetylase 1; NSL, non-specific-lethal; MOF, males absent on the first; GSK3β, Glycogen Synthase Kinase 3β; STAT3, signal transducer and activator of transcription 3; AKT, protein kinase B; Polη, DNA polymeraseη; DM, diabetes mellitus; AD, Alzheimer’s Disease.
